# Fidanacogene Elaparvovec Gene Therapy: Budesonide Treatment of a Late Decline in Factor IX Expression in a Patient with Hemophilia B

**DOI:** 10.3390/ijms27209049

**Published:** 2026-10-12

**Authors:** Kerstin Herbst, Behnaz Pezeshkpoor, Claudia Klein, Thilo Albert, Philipp Lutz, Christian Strassburg, Ulrich Spengler, Georg Goldmann, Johannes Oldenburg

**Affiliations:** 1 Institute of Experimental Hematology and Transfusion Medicine, University of Bonn, 53127 Bonn, Germany; kerstin.herbst@ukbonn.de (K.H.); behnaz.pezeshkpoor@ukbonn.de (B.P.); claudia.klein@ukbonn.de (C.K.); thilo.albert@ukbonn.de (T.A.); georg.goldmann@ukbonn.de (G.G.); 2 Center for Rare Diseases Bonn (ZSEB), University Clinic Bonn, 53127 Bonn, Germany; 3 Department of General Internal Medicine I, University of Bonn, 53127 Bonn, Germany; philipp.lutz@ukbonn.de (P.L.); christian.strassburg@ukbonn.de (C.S.);

**Keywords:** alanine transaminase, gene therapy, hemophilia B, treatment outcome, fidanacogene elaparvovec

## Abstract

Fidanacogene elaparvovec is an adeno-associated virus (AAV)-based gene therapy that enables endogenous factor IX (FIX) expression in hemophilia B, potentially eliminating the need for prophylactic FIX replacement. Post-treatment monitoring focuses on FIX durability, hepatic safety, and immune-mediated loss of expression. A 40-year-old man with severe hemophilia B received a single intravenous dose of fidanacogene elaparvovec in the phase 3 BENEGENE-2 trial (NCT03861273). FIX activity was assessed by one-stage and chromogenic assays. Liver enzymes, clinical parameters, and adverse events were monitored. Prednisolone was initiated for liver enzyme elevation and later switched to budesonide in response to declining FIX levels. Follow-up continued for 4 years. FIX activity peaked at week 6 (~30 IU/dL) and remained stable until week 16. Moderate ALT elevation (peak ~70 U/L) responded to prednisolone. Between weeks 22–28, FIX levels declined without ALT elevation. Budesonide (9 mg/day) was started at week 25 for 3 months and gradually tapered, stabilizing FIX activity at 10-20 IU/dL. This level has been maintained for 4 years without bleeding episodes or FIX replacement. Fidanacogene elaparvovec provided sustained, clinically meaningful FIX expression. Budesonide may offer an effective hepatocyte-targeted alternative to systemic corticosteroids while supporting long-term transgene expression.

## 1. Introduction

Hemophilia B is an X-linked inherited bleeding disorder caused by mutations in the *F9* gene, leading to a deficiency or dysfunction of coagulation factor IX (FIX). The severity of hemophilia B is classified based on plasma FIX activity: severe (<1 IU/dL), moderate (1–5 IU/dL), and mild (>5–<40 IU/dL) [1]. Patients with severe hemophilia B are at high risk of spontaneous and trauma-related bleeding episodes, including life-threatening hemorrhages and progressive chronic joint damage due to recurrent hemarthroses.

The standard treatment consists of intravenous FIX replacement therapy, administrated either on-demand or as prophylaxis. While this approach is effective in preventing bleeding, it is associated with regular intravenous injections, placing a substantial burden on patients. Thus, current therapies remain non-curative and do not eliminate the lifelong need for regular treatment. In this context, gene therapy has emerged as a transformative approach for hemophilia B, offering the potential for sustained endogenous FIX expression following a single administration. Among the most promising platforms are adeno-associated virus (AAV)-based vectors, which deliver a functional *F9* transgene to hepatocytes under liver-specific promoters. Clinical trials have shown that AAV-mediated gene transfer can achieve long-term therapeutic FIX levels, potentially eliminating the need for regular prophylaxis and reducing bleeding rates to near zero [2].

Fidanacogene elaparvovec is an investigational AAV-based gene therapy developed for hemophilia B. It uses an AAV Rh74var capsid to deliver a codon-optimized cDNA encoding the hyperactive FIX-Padua variant, which has about 8-fold higher specific activity than wild-type FIX. The transgene is expressed under a liver-selective promoter, and the vector is designed to drive therapeutic FIX levels with a favorable safety profile. Clinical trials have reported durable FIX expression in most treated patients, though interindividual variability and late declines in FIX levels have been observed [3].

A known challenge of AAV-based gene therapy is the risk of immune responses to the viral capsid or transduced hepatocytes, potentially leading to elevations in liver enzymes (e.g., alanine transaminase (ALT)) and loss of transgene expression [4]. To mitigate these immune responses, corticosteroids such as prednisolone are commonly used during the post-infusion period. However, systemic steroids can be associated with significant side effects, especially when prolonged therapy is needed. Budesonide, a corticosteroid with high first-pass hepatic metabolism, offers a potential alternative for immunosuppression targeting liver-specific inflammation while reducing systemic exposure [5]. Its use has been well established in inflammatory liver conditions, such as autoimmune hepatitis [6], but remains underexplored in the context of gene therapy.

Here, we present a case of a male patient with severe hemophilia B who was part of this phase 3 trial with fidanacogene elaparvovec. Our patient showed a late decline in FIX expression between weeks 22 and 28, which was treated with budesonide, probably resulting in a long-term stable expression of FIX. This report offers insights into personalized immune modulation strategies for sustaining FIX expression after gene therapy and highlights differences in durability and immune management.

## 2. Results

### 2.1. Early FIX Expression and Liver Enzyme Dynamics

**Figure 1:**
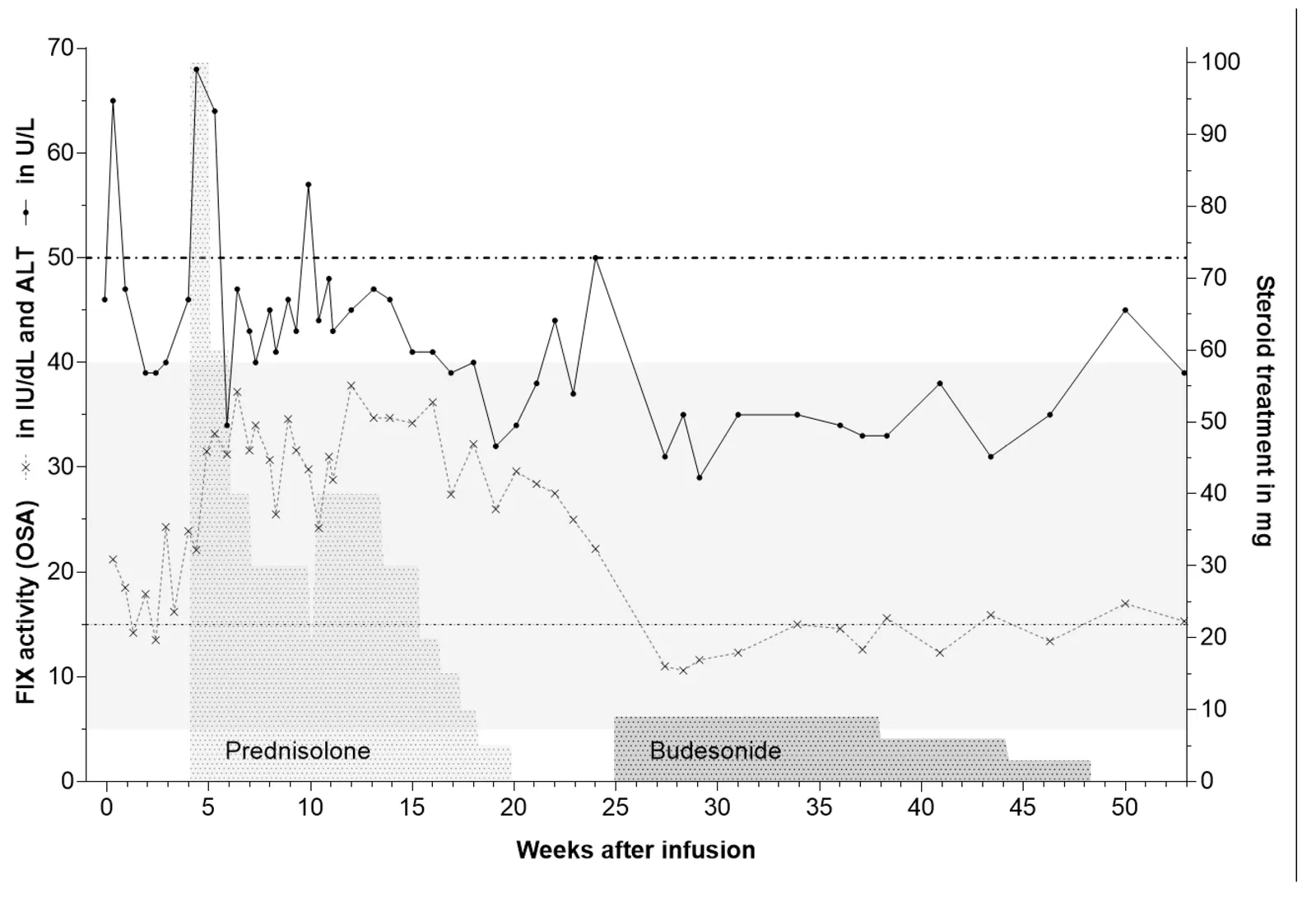
FIX activity, ALT levels and immune suppression in the first year after fidanacogene elaparvovec infusion. Initial immunosuppression consisted of 100 mg prednisolone daily, followed by a tapering regimen until week 20. Budesonide was introduced at week 25 at a dose of 3 mg three times daily. Tapering began at week 38, with the dose reduced to 3 mg twice daily and subsequently to 3 mg once daily. The course of ALT (U/L, circles with solid line) and FIX:C (IU/dL, OSA_Actin FS_, crosses with dotted line) measurements over the first year is shown, with both plotted on the left y-axis. Steroid treatment doses (prednisolone and budesonide) are plotted on the right y-axis. The black dotted line indicates the upper limit of normal of ALT (50 U/L). The grey shaded area corresponds to FIX activity levels in mild hemophilia (5–40 IU/dL), with the grey dotted line marking 15 IU/dL as the threshold for preventing spontaneous bleeding [7].

In the initial weeks following fidanacogene elaparvovec infusion, the patient demonstrated a robust increase in FIX activity, reaching peak levels of approximately 37 IU/dL at week 6, as measured by one-stage assay (OSA_Actin FS_) (Figure 1). This initial response confirmed successful hepatic transduction and active transgene expression of the FIX-Padua variant.

During the first 16 weeks post-infusion, FIX levels remained stable and above prophylactic thresholds (>15 IU/dL), and no bleeding episodes were reported. Concurrently, ALT levels showed only mild-to-moderate elevation, with a maximum of 68 U/L (baseline: 44 U/L), not exceeding grade 1 hepatotoxicity. Per protocol, the patient received oral prednisolone (initially 100 mg/day) starting at week four after gene transfer. The dose was gradually tapered until complete discontinuation by week 20.

### 2.2. Decline in FIX Activity and Immune Suppressive Therapy

At week 25, a progressive decline in FIX activity was observed, with a drop of more than 60% from about 30 IU/dL in week 22 to 11 IU/dL in week 28 (Figure 1). There was a mild ALT rise (1.14 above baseline) associated with the decrease in FIX activity.

To address the declining FIX levels and avoid systemic steroid exposure, oral budesonide (9 mg/day) was initiated at week 25, targeting localized hepatic immunosuppression. This intervention stopped the progressive decline in FIX activity and led to stabilization of FIX activity between 11.0–18.4 IU/dL (OSA_Actin FS_, median 13.4 IU/dL; *n* = 13 by month 12). FIX remained stable until now for more than 4 years. Budesonide was gradually tapered to 6 mg/day at week 36, and then maintained at 3 mg/day until week 48. No side effects of budesonide treatment were observed. ALT levels stayed within the upper normal range during this period, and the patient remained free of bleeding symptoms without the need for FIX replacement therapy.

### 2.3. Long-Term Follow-Up and Durability of FIX Expression

**Figure 2:**
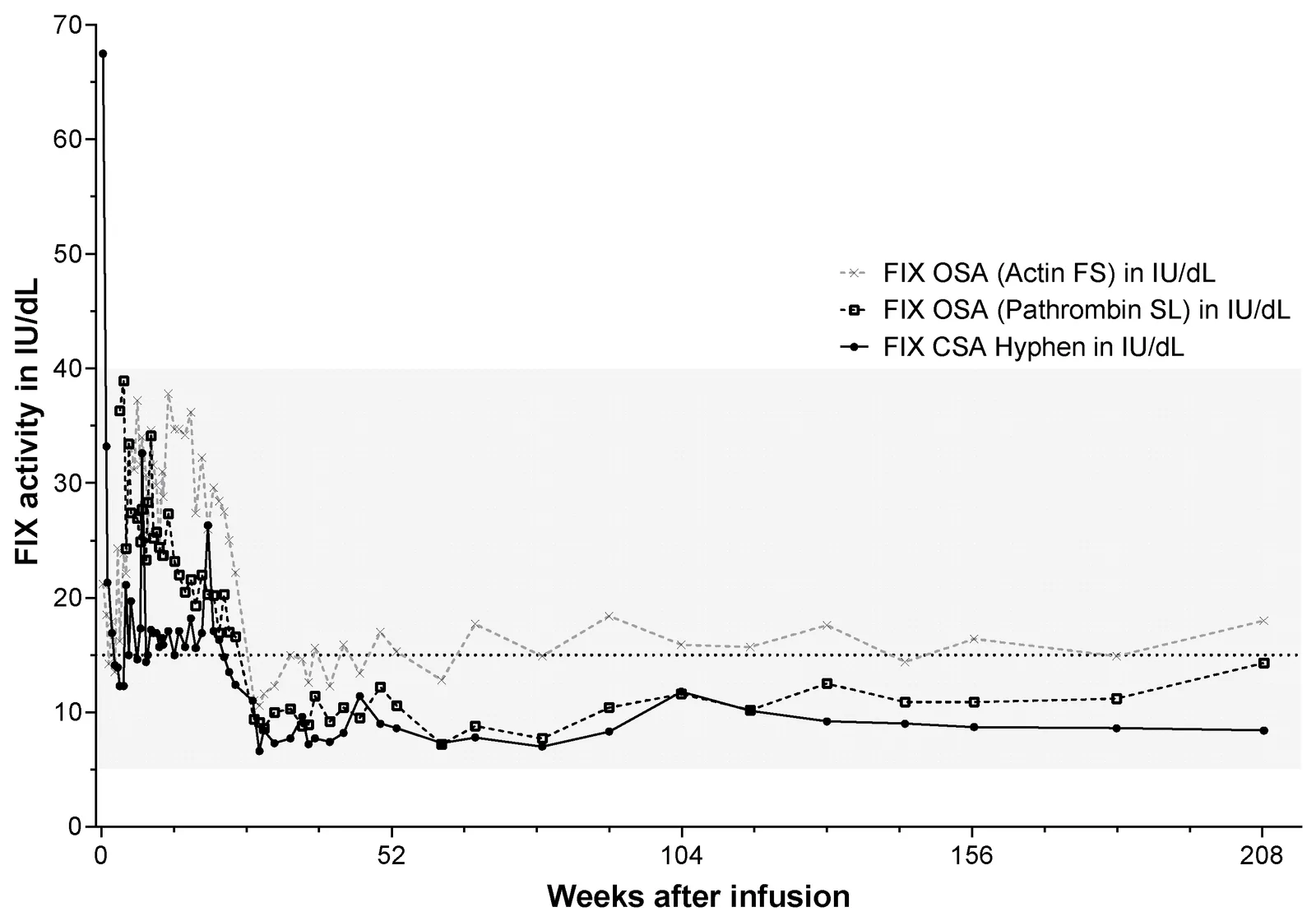
FIX activity levels over four years of follow-up. FIX:C was measured by OSA with Actin FS (crosses with dotted line) and Pathrombin SL (boxes with dotted line), and CSA (circles with solid line), the results of which are blotted on the y-axis. The grey shaded area corresponds to FIX activity levels in mild hemophilia (5–40 IU/dL), with the dotted line marking 15 IU/dL as the threshold for preventing spontaneous bleeding [7].

Longitudinal monitoring of FIX activity over a period of 4 years demonstrated sustained endogenous expression following a single infusion of fidanacogene elaparvovec (Figure 2). After the initial decline in FIX levels between weeks 22 and 28, the patient’s FIX activity plateaued and remained stable. To quantify FIX expression, three different activity assays were used.

All FIX assays demonstrated consistent results, with median FIX activity levels indicating mild hemophilia B severity during long-term follow-up (median values from week 53 to week 182: FIX:C by CSA, 8.6 IU/dL [*n =* 11]; by OSA_Actin FS_, 15.3 IU/dL [*n* = 11]). The OSA values consistently yielded 1.8-fold higher FIX activity compared to CSA, a well-documented phenomenon in gene therapy monitoring due to assay-specific differences in sensitivity to the hyperactive FIX-Padua variant [8]. There was no further decline in FIX expression in 4 years of observation time. The patient remained free of bleeding events and did not require FIX replacement therapy throughout the entire observation period. Liver enzymes remained within normal ranges, and no additional immunosuppressive therapy was required after tapering of budesonide at week 48.

### 2.4. Safety Profile and Adverse Events

The patient experienced a total of 12 adverse events (AEs) during the follow-up period after administration of fidanacogene elaparvovec (Table 1). None of the AEs were classified as serious adverse events (SAEs). Most AEs were mild in severity, with only one severe episode of fatigue (flabbiness/astenia) occurring shortly after gene therapy, which resolved without intervention.

During the follow-up, three episodes of ALT elevation were documented, with a baseline ALT of 44 U/dL. Only the elevation at week 4 exceeded the critical threshold of 1.5-fold above baseline and was treated with oral corticosteroids. Mild LDH and creatinine elevations were observed transiently and were attributed to concomitant drug use or unrelated causes.

A SARS-CoV-2 infection was recorded in week 24, categorized as mild. A common cold episode at week 34 was classified as moderate and self-limiting. One case of contact dermatitis was considered unrelated to gene therapy and linked to drill milk exposure. No infusion reactions, anaphylaxis, development of FIX inhibitors, thrombotic events, or malignancies were observed throughout the 4 year follow-up. Importantly, no spontaneous or trauma-related bleeding events occurred, consistent with the sustained FIX activity observed.

### 2.5. Clinical Outcome

By the end of the more than 4-year follow-up, the patient demonstrated sustained FIX activity levels of about 15 IU/dL FIX:C with OSA_Actin FS_ without any bleeding episode requiring FIX infusions. These findings demonstrate that despite a decline at month 5, FIX expression following fidanacogene elaparvovec infusion can reach a stable plateau, enabling bleed protection comparable to mild hemophilia B and long-term prophylactic benefit.

## 3. Discussion

Gene therapy with fidanacogene elaparvovec offers the potential to eliminate the need for regular FIX prophylaxis and reduce treatment burden in hemophilia B [9,10]. We present a patient who achieved sustained clinical benefit for more than 4 years, without bleeding events or additional FIX replacement therapy. These results highlight the potential for long-term efficacy and safety of AAV-based gene therapy in real-world settings [11].

In our patient, FIX activity plateaued between 10 and 20 IU/dL, with a median FIX:C of 15.3 IU/dL measured by the OSA_Actin FS_ (*n* = 11; weeks 53–182). This level was sufficient to prevent both trauma-related and spontaneous bleeding episodes. The durability of FIX expression is consistent with findings from long-term follow-up of fidanacogene elaparvovec trials, in which median FIX:C measured by OSA was 22.9% at month 15 (*n* = 38) and 22.6% after 4 years (*n* = 10) [3,12]. However, inter-individual variability and late declines in FIX activity have been observed. In the phase 3 study, six patients resumed FIX prophylaxis within 15 months after fidanacogene elaparvovec infusion [3,13]. All had experienced ALT elevations and received corticosteroid treatment. Three of these patients subsequently developed a late decline in FIX:C more than 4 months after treatment, despite having already established substantial endogenous FIX:C levels [3,13].

In the phase 3 trial of fidanacogene elaparvovec, 53% of patients developed ALT elevations, most of which were managed with systemic corticosteroids [3]. Following gene therapy, our patient initially demonstrated robust FIX expression and developed an early ALT elevation that was successfully managed with systemic prednisolone in accordance with the study protocol. A subsequent decline in FIX activity led to the initiation of budesonide rather than re-escalation of systemic corticosteroids, coinciding with a mild rise in ALT levels at week 25. Budesonide was selected because of its potent local anti-inflammatory activity in hepatocytes and extensive first-pass hepatic metabolism [5]. It is also an established treatment for autoimmune hepatitis [14], providing a biological rationale for its use in this setting. FIX activity subsequently remained stable for more than 4 years.

Importantly, observations from other participants in the previously published fidanacogene elaparvovec cohort suggest that declines in FIX activity may occur independently of the magnitude of ALT elevation [3]. Participant 11, for example, showed a decline in FIX activity from 26% to 10% despite near-baseline ALT/AST levels, followed by a further decline to 5.2% and 4.2% in the absence of ALT elevation. Similarly, Participant 23 experienced a decline from 17% to 6% despite stable liver enzyme levels, while Participant 36 showed a decline from 33% to 5.7% despite only mildly elevated ALT (54 U/L). All three patients ultimately resumed FIX prophylaxis because of complete loss of exogenous FIX activity. These observations suggest that FIX kinetics following gene therapy are not solely determined by the degree of biochemical evidence of hepatocellular inflammation and that clinically relevant declines in transgene expression may occur even in the absence of substantial ALT elevations.

The available data do not exclude that FIX activity might have spontaneously recovered in the absence of additional immunosuppression with budesonide. However, the late complete loss of expression in three patients in the phase 3 trial of fidanacogene elaparvovec suggests a significant risk of expression failure. In our patient, budesonide was initiated in the setting of a clinically relevant decline in FIX activity. A potential benefit of budesonide had previously been observed in a patient undergoing hemophilia A gene therapy at our center [15]. In the present case, the subsequent long-term stability of FIX expression raises the possibility that hepatoselective immunosuppression with budesonide contributed to maintaining transgene expression. However, given the single-patient nature of this observation, causality cannot be proved with certainty.

Late declines in FIX expression have also been reported with other AAV-based FIX gene therapy approaches. In the dose-escalation study by Chowdary et al., all patients received prophylactic immunosuppressive therapy with corticosteroids, with or without tacrolimus. Despite initial normalization or supraphysiologic FIX levels, six of ten patients experienced a late decline in FIX expression, and one patient resumed FIX prophylaxis [16]. Potential explanations included cessation of immunosuppressive therapy and higher vector genome doses [16,17]. Etranacogene dezaparvovec (Hemgenix^®^) demonstrated higher median FIX expression of approximately 40 IU/dL and a lower incidence of clinically relevant FIX decline, with a reduction of more than 20% observed in 17% of patients after 1 year [18]. Treatment failure occurred in isolated cases associated with infusion reactions or pre-existing neutralizing antibodies, and one patient resumed FIX prophylaxis [18,19]. Taken together, clinical trials of AAV-based FIX gene therapy using the FIX-Padua variant have demonstrated durable FIX expression, although substantial inter-individual variability and late declines in FIX activity remain evident [3,18].

The mechanisms underlying late declines in FIX expression remain incompletely understood. Importantly, the available clinical observations suggest that such declines may not always be accompanied by marked ALT elevations, challenging the assumption that transgene loss is exclusively driven by overt hepatocellular inflammation. Whether these late declines reflect immune-mediated clearance of transduced hepatocytes, other vector-related mechanisms, or inter-individual differences in vector biology remains uncertain [16,17]. Consequently, the relationship between ALT dynamics, immunosuppressive treatment, and long-term FIX expression warrants further investigation.

Another important challenge is the interpretation of FIX activity following gene therapy. Published data have shown that linking FIX activity levels directly to clinical outcomes is difficult, largely because bleeding events are infrequent above certain activity thresholds. Nevertheless, a correlation between OSA-measured FIX levels and joint health has been reported [8]. In our patient, FIX levels were measured using OSA_Actin FS_, OSA_PSL_, and CSA assays to provide a comprehensive profile. OSA consistently yielded higher FIX activity values than CSA, reflecting a recognized issue in the assessment of FIX-Padua, which has altered specific activity and assay reactivity compared with wild-type FIX [20,21]. Despite recognition of this issue, no assay has yet been standardized or specifically validated for FIX-Padua, limiting the interpretation of individual FIX values and complicating comparisons across studies.

This underscores the need for standardized Padua-sensitive assays for both clinical monitoring and research. In particular, assay harmonization will be important for defining clinically meaningful thresholds, identifying true late declines in FIX expression, and comparing outcomes across different AAV-based gene therapy platforms.

Taken together, our case demonstrates durable clinical efficacy of fidanacogene elaparvovec, with sustained FIX activity and complete protection from bleeding for more than 4 years. The use of individualized immunosuppression, including hepatoselective treatment with budesonide, coincided with stabilization of FIX expression during a late decline in FIX activity and allowed avoidance of prolonged systemic corticosteroid exposure. Although this observation raises the possibility that budesonide may help stabilize transgene expression in patients with mild ALT elevations and emerging FIX decline, a causal relationship cannot be established from a single case. The number of reported cases remains too limited to determine its efficacy, optimal timing, or appropriate patient selection. Further systematic data are therefore needed to assess whether early hepatoselective immunosuppression can influence the long-term stability of FIX expression following AAV-based gene therapy.

## 4. Materials and Methods

Patient Description: A 40-year-old male with severe hemophilia B (FIX activity < 1 IU/dL) due to a splice site pathogenic variant in *F9* (NM_000133, c.787+3 A>C) was treated with gene therapy using fidanacogene elaparvovec as part of a clinical trial (BENEGENE-2, NCT03861273). His medical history included hemophilic arthropathies in the right ankle and left knee, and resolved hepatitis B and C infections, but no other clinically relevant comorbidities. Liver ultrasound and laboratory tests confirmed a healthy hepatic profile at baseline. Prior to gene therapy, the patient was receiving prophylactic treatment with recombinant FIX (Nonacog beta pegol, Novo Nordisk A/S, Bagsværd, Denmark), administered at a dose of approximately 30 IU/kg weekly. The FIX replacement therapy was discontinued following vector administration. The patient received a single intravenous infusion of fidanacogene elaparvovec at a dose of 5 × 10^11^ vg/kg. The infusion was well tolerated without any acute adverse events. Liver function tests and FIX activity levels were monitored longitudinally per protocol.

Coagulation parameter assessments: For measuring FIX activity (FIX:C), citrated plasma was prepared by centrifugation of peripheral blood samples at 2500× *g* for 15 min. Samples were analyzed fresh or stored at ≤−40 °C in aliquots until testing. FIX activity was measured using a Sysmex CN-6000 system (Siemens Healthineers, Forchheim, Germany) with two different assay types. Two one-stage clotting assays (OSA) were performed, based on either Actin FS (OSA_Actin FS_) or Pathrombin SL reagents (OSA_PSL_) (Siemens Healthineers). In addition, a chromogenic substrate assay (CSA) was conducted using the BIOPHEN™ FIX kit (CoaChrom Diagnostica GmbH, Neuville-sur-Oise, France). All assays were calibrated against standard human plasma (SHP; Siemens Healthineers) [22]. FIX inhibitors were monitored as previously described with modifications [23,24]. Liver enzymes, including gamma-glutamyl transferase (GGT), alanine transaminase (ALT) and aspartate aminotransferase (AST) levels, were determined in plasma in units per liter (U/L) on a Cobas^®^ c702 (Roche Diagnostics, Basel, Switzerland) using the ASTPM reagents (Roche Diagnostics). Reference values provided by the laboratory were as follows: GGT, < 60 U/L; ALT, < 50 U/L; and AST, < 50 U/L.

## Figures and Tables

**Table 1:** Overview of the patient’s adverse events experienced during the observation period. AE, adverse event; ALT, alanine transaminase; SAE, serious AE.

		Year 1 (Until Week 52)	Years 2–4 (Until Week 208)
AEs		5	0
SAEs		0	0
Treatment-related AEs		5	0
Glucocorticoid-related AEs		2	0
AEs of special interest	ALT elevation	3	0
	ALT elevation grade 3	0	0
	Infusion-related reaction	0	0
	Anaphylactic/anaphylactoid reactions	0	0
	Thromboembolic events	0	0
	Any malignancy	0	0

## Data Availability

The original contributions presented in this study are included in the article. Further inquiries can be directed to the corresponding author.

## Abbreviations

The following abbreviations are used in this manuscript:

AAV	Adeno-Associated Virus
AE	Adverse Event
ALT	Alanine Transaminase
AST	Aspartate Aminotransferase
CSA	Chromogenic Substrate Assay
FIX	Factor IX
FIX:C	Factor IX Coagulant Activity
F9	coagulation factor IX gene
GGT	Gamma-Glutamyl Transferase
IU/dL	International Units per deciliter
LDH	Lactate Dehydrogenase
NCT	National Clinical Trial (registration identifier)
OSA	One-Stage Assay
OSA_Actin FS_	One-Stage Assay (Actin FS reagent)
OSA_PSL_	One-Stage Assay (Pathrombin SL reagent)
SAE	Serious Adverse Event
SHP	Standard Human Plasma
SARS-CoV-2	Severe Acute Respiratory Syndrome Coronavirus 2
U/L	Units per Liter
vg/kg	Vector Genome per Kilogram (body weight)
